# The Impact of COVID-19 on Cancer Care in the Post Pandemic World: Five Major Lessons Learnt from Challenges and Countermeasures of Major Asian Cancer Centres

**DOI:** 10.31557/APJCP.2021.22.3.681

**Published:** 2021-03

**Authors:** Laureline Gatellier, Abhishek Shankar, Luh K Mela Dewi, Quazi Mushtaq Hussain, Tashi Dendup Wangdi, Dato Babu Sukumaran, Nina Kemala Kemala Sari, Sahar Tavakkoli Shiraji, Mohammad Biglari, Mamak Tahmasebi, Satoshi Iwata, Tatsuya Suzuki, Seung-Kwon Myung, June Young Chun, Jong Soo Han, Fen Nee Lau, Suhana Yusak, Luvsandorj Bayarsaikhan, Khin Thin Mu, Kishore K. Pradhananga, Aasim Yusuf, Ching-Hung Lin, Ruru Chun-Ju Chiang, Suleeporn Sangrajran, Quang Tien Nguyen, Giang Nguyen Huong, Aung Naing Soe, D N Sharma, Manju Sengar, C S Pramesh, Tomohiro Matsuda, Alireza Mosavi jarrahi, William Hwang

**Affiliations:** 1 *National Cancer Center, Tokyo, Japan. *; 2 *Lady Hardinge Medical College & Associated Hospitals, Delhi, India. *; 3 *Dharmais Hospital - National Cancer Center, Jakarta, Indonesia. *; 4 *National Institute of Cancer Research and Hospital, Dhaka, Bangladesh.*; 5 *JDWNR Hospital Thimphu University of Medical Sciences, Bhutan. *; 6 *The Brunei Cancer Centre, Brunei Darussalam. *; 7 *Hematology, Oncology and Bone Marrow transplantation Research Center, Shariati Hospital, Tehran University of Medical Science,Tehran, Iran. *; 8 *Cancer Institute, Tehran University of Medical Sciences, Tehran, Iran. *; 9 *National Cancer Center of Korea, Goyang-si Gyeonggi-do, Republic of Korea. *; 10 *National Cancer Institute, Putrajaya, Malaysia. *; 11 *National Cancer Center of Mongolia, Ulaanbaatar, Mongolia. *; 12 *Myanmar Yangon General Hospital, Yangon, Myanmar. *; 13 *Kathmandu Cancer Center, Bhaktapur, Nepal. *; 14 *Shaukat Khanum Memorial Cancer Hospital & Research Centre, Lahore, Pakistan. *; 15 *National Taiwan University Cancer Center Hospital, Taipei City, Taiwan. *; 16 *Taiwan Cancer Registry, and Institute of Epidemiology and Preventive Medicine, College of Public Health, National Taiwan University, Taipei, Taiwan. *; 17 *National Cancer Institute, Bangkok, Thailand. *; 18 *National Cancer Institute, National Cancer Hospital, Hanoi, Vietnam. *; 19 *City Cancer Challenge Foundation, Geneva, Switzerland. *; 20 *Dr BR Ambedkar Institute Rotary Cancer Hospital & National Cancer Institute, All India Institute of Medical Sciences, Delhi, India. *; 21 *Tata Memorial Centre, Homi Bhabha National Institute, Mumbai, Maharashtra, India. *; 22 *Medical School, Shahid Behshti University of Medical Sciences, Tehran, Iran. *; 23 *National Cancer Centre, Singapore. *

**Keywords:** Asia, Coronavirus, prevention, diagnosis, LMIC

## Abstract

**Objective:**

The COVID-19 pandemic has dramatically affected healthcare services around Asia. The Asian National Cancer Centres Alliance and the Asia-Pacific Organisation for Cancer Prevention collaborated to assess the mid- and long- term impact of COVID-19 to cancer care in Asia.

**Methods:**

The two entities organised a combined symposium and post-meeting interactions among representatives of major cancer centres from seventeen Asian countries to outlining major challenges and countermeasures.

**Results:**

Participating stakeholders distilled five big questions. 1) *“Will there be an explosion of late-stage cancers after the pandemic?” *To address and recover from perceived delayed prevention, screening, treatment and care challenges, collaboration of key stakeholders in the region and alignment in cancer care management, policy intervention and cancer registry initiatives would be of essential value. 2) *“Operations and Finance”* The pandemic has resulted in significant material and financial casualties. Flagged acute challenges (shortages of supplies, imposition of lockdown) as well as longer-standing reduction of financial revenue, manpower, international collaboration, and training should also be addressed. 3) *“Will telemedicine and technological innovations revolutionize cancer care?”* Deploying and implementing telemedicine such as teleconsultation and virtual tumour boards were considered invaluable. These innovations could become a new regular practice, leading to expansion of tele-collaboration through collaboration of institutions in the region. 4) *“Will virtual conferences continue after the pandemic?”* Virtual conferences during the pandemic have opened new doors for knowledge sharing, especially for representatives of low- and middle-income countries in the region, while saving time and costs of travel. 5) *“How do we prepare for the next pandemic or international emergency?”* Roadmaps for action to improve access to appropriate patient care and research were identified and scrutinised.

**Conclusion:**

Through addressing these five big questions, focused collaboration among members and with international organisations such as City Cancer Challenge will allow enhanced preparedness for future international emergencies.

## Introduction

The COVID-19 pandemic has affected health care services in many dimensions: from interruption of regular patient flow to cancer care facilities and the overwhelming of health care resources, to the implementation of extra protective measures and social distancing with increased utilization of telehealth services (Jazieh et al., 2020). Additionally, despite cancer patients need continuous care and cannot afford to have deferment of diagnostic tests or therapeutic interventions, oncologists around the world have started changing their daily practice to limit cancer patients’ exposure to COVID-19, potentially very risky or even fatal (Moujaess et al., 2020). 

As a precautionary approach, oncology practices implemented specific measures such as reducing the number of patients in outpatient clinics, reducing unnecessary or elective procedures, and discharging patients from inpatient services (Jazieh et al., 2020). However, these measures have posed major challenges to oncologists who have needed to balance the delivery of high-quality continuous unfragmented cancer care while minimizing the risk of exposure of patients to COVID-19 during care. 

The negative impact of the pandemic was particularly acute in low and middle-income countries (LMIC) with limited resources, poor infrastructure, shortage of health care providers and organized care teams, scarcity of medical supplies and personal protective equipment (PPE), and poor access to technology leading to inability in providing and delivering optimal care (Basu, 2020; Bouanani et al., 2020; Faizal et al., 2020; McMahon et al., 2020). 

This pandemic has exposed the weaknesses and lack of resilience of healthcare systems and these flaws have had major consequences for cancer care and cancer patients (Raymond et al., 2020). Crucial attention is needed, not only to restore cancer care delivery but to build improved systems, processes and infrastructure as long-term norm and to be better prepared to fight the next crisis. 

Multiple medical organisations and cancer centres have come up with recommendations for the management of different cancer types and safety standards in continuing cancer treatment in the patients with COVID-19, highlighting numerous critical issues in maintaining the clinical standards for cancer treatments during this period of declining resources - leading to a real humanitarian crisis (Singh and Prasad, 2020; Akladios et al., 2020; Coles et al., 2020; Fakhry et al., 2020; Fix et al., 2020; Ganne-Carrie et al., 2020; Head et al., 2020; Lou et al., 2020; Mian et al., 2020; Mohile et al., 2020; Percival et al., 2020; Ramakrishna et al., 2020; Ramirez et al., 2020; Thoracic Surgery Outcomes Research Network et al., 2020; Yahalom et al., 2020; Zic et al., 2020). 

The current strategies taken by major cancer centres in different countries are likely to evolve over time with evolving priorities to treat cancer patients while ensuring protection of both patients and health care workers. The Asian National Cancer Centres Alliance (ANCCA) published an Asian perspective on COVID-19 management with a regional comparison to address challenges in cancer care delivery in Asia in August 2020 (Dewi et al., 2020; Gatellier et al., 2020). As the responses of oncology centres to the pandemic and interventions implemented were based on data available on the early stage of the pandemic at National Cancer Centres, the Asian National Cancer Centres Alliance (ANCCA) and Asian Pacific Organization for Cancer Prevention (APOCP) expanded this work through the collaboration on the Impact of COVID-19 on Cancer and decided to bring oncologists, researchers, and policy makers from 17 countries in Asia to precisely delineate the challenges that COVID-19 has posed for cancer care delivery, as well as to amplify activities and efforts to address these challenges, and produce recommendations on the most critical and urgent cancer care needs. 

Our Network perceives that both ANCCA and APOCP can play a crucial supportive role to regional and national health systems, through provision of timely and targeted advice, guidance, and coordination. To address cancer care delivery amidst COVID-19 crisis, representatives from major cancer centres in 17 countries participated in this collaborative initiative through a half day virtual symposium followed by in-depth information sharing (Hwang et al., 2021) and presented collective challenges (Table 1) and countermeasures (Table 2) for continuing cancer care during the pandemic. Challenges and countermeasures included addressing the disruption of cancer care delivery, managing the backlog of patients after the lockdown, restoring the confidence of patients on the safety of cancer treatment, ensuring uninterrupted supply of medicines, products and equipment, addressing cancer workforce gaps across the Asian continent, as well as employing innovative technologies and solutions to strengthen cancer systems and provide optimal care to cancer patients, and securing long-term collaboration of cancer centres in Asia. From the many issues highlighted, ANCCA and APOCP distilled five major questions on that we will need to address in this post pandemic world.

*Will there be an explosion of late-stage cancer after the pandemic?*

The COVID-19 pandemic posed critical challenges to current oncology care and practices including late diagnoses, delayed anti-cancer treatment, and stalled clinical trials (Ray et al., 2020). In response, healthcare systems are rapidly reorganizing cancer services to ensure that patients continue to receive essential care while minimizing risk of exposure to SARS-CoV-2 infection.

Most of the cancer centres in our collaborative initiative (14 out of 17) reported a decrease in number of cancer patients during the pandemic except Brunei, Iran and Taiwan (Table 1). This could be due to a decrease or deferment in follow up appointments, but as COVID-19 continues to impact nearly all aspects of cancer care, most of the countries also saw a troubling drop in new cancer diagnoses since the pandemic began. This observed drop follows a similar tendency with the 46% decrease in the diagnoses of the six most common cancer types - breast, colorectal, lung, pancreatic, gastric and oesophageal cancers during the COVID-19 pandemic at Dana Farber Cancer Institute (Bakouny et al.). Similar situation was observed in European Union (van de Haar et al., 2020), United States of America (Lou et al., 2020), United Kingdom (Greenwood and Swanton, 2021), Saudi Arabia (Alessy et al., 2020), United Arab Emirates (Al-Shamsi et al., 2020), Greek (Miltiadou et al., 2020), India (Kumar et al., 2020), and Morocco (Bouanani et al., 2020) . Whether this will result in an increase in late stage diagnoses remains to be confirmed with time.

This pandemic also made a toll on mental health of cancer patients and caregivers and has been shown in various studies across the globe (Ng et al., 2020). This includes the impacts of social isolation, family disruption, and occupational and financial challenges. Brunei reported clinicians’ observations of an increase in fear and anxiety among patients resulting in less clinical consultations during the initial phase of the pandemic, even though cancer treatment services continued to run without any disruption. Unlike other institutions, the cancer centres from Korea, Malaysia and Taiwan did not report significant difficulty or delays in continuing cancer care (Table 1 “Difficulty or delay to procure cancer treatment”). 

Cancer prevention and screening services are important components of national cancer strategies, but these were considered as non-essential during the crisis. During this pandemic, approximately 65% institutions in our survey (11 out of 17) delayed or suspended their cancer prevention and screening services to prevent the transmission of coronavirus infection. For example, NCC Indonesia reported that outpatient visits to cancer screening and diagnostic procedures in NCC Indonesia were respectively 59% and 35% lower in 2020 in comparison to 2019. This in tandem with what happened globally as, during the early stages of the pandemic, major medical professional organisations such as the U.S. Center for Disease Control and Prevention recommended that cancer screening and other health prevention services should be postponed unless the risks outweighed the benefits. By contrast, as the Korean government did not restrict its screening program because of social distancing, a more limited decrease in screening was observed (8%) between the year 2019 and 2020 in NCC Korea.

As hospitals prepared to meet the challenges from the COVID-19 pandemic, cancer patients and individuals enrolled in screening and early detection programs were unable to access appropriate care in this pandemic (Spagnoletti et al., 2020). Cancer screening and early detection services were suspended in many countries, with uncertainty on how quickly the backlog could be cleared (Bakouny et al., 2020). Asian citizens with potential symptoms of cancer have also delayed from seeking medical advice due to fears of being infected. As screening helps to detect cancer at early stage, deferment could result in a rise in late-stage cancer with a corresponding increase in cancer-related mortality and morbidity. A national, population-based modelling study in the UK predicts that the COVID-19 pandemic will result in up to 9.6%, 16.6%, 5.3%, and 6.0% of additional deaths due to breast, colorectal, lung, and oesophageal cancer respectively up to 5 years after diagnosis and that urgent policy intervention are necessary to limit the impact of the COVID-19 pandemic on patients with cancer (Maringe et al., 2020). 

An increased load of patients in cancer care facilities is already being observed among participating institutions (Table1 “Increase of patients after the pandemic”) and a further rise is expected in later months this year with increased screening, diagnosis, treatment initiation or other outpatient and inpatient visits (Shankar et al., 2021). Based on information extracted from our collaborative initiative (Hwang et al., 2021), as well as various reports received from patient organizations and healthcare professionals across Asia, the harmful impact to cancer services resulting from the COVID-19 pandemic has occurred and is expected to last across the entire cancer care continuum if actions in the region are not deeply considered (Barbhuiya, 2020). 

As a result of the pandemic, the collaborative initiative has underlined pre-existing inequalities in cancer and healthcare have been widened (Hwang et al., 2021). One example is the temporary supply chain disruption (including medical equipment and chemotherapy drugs in Bangladesh, India, Indonesia, Iran, Japan, Malaysia, Myanmar, and Singapore (Table 1, “Shortage of Supply”), which has likely even further impacted care of cancer patients in LMICs. Additionally, delays in cancer prevention, screening and diagnosis (Table 1 “Delay or suspension of cancer prevention, detection and screening”) are likely to lead to increased presentations with advanced disease and poorer prognosis, in which case patients and their families are faced with more complex decision-making because of resource constraints. Furthermore, decreased availability of outside support has placed a significantly increased burden on caregivers, left alone to take care of cancer patients. Altogether, these elements are exacerbating psycho-social distress and a sense of powerlessness, highlighting an urgent need for the promotion of psychosocial care of cancer patients, caregivers, and families during the pandemic (Table 1, “Fear and Anxiety of patient or Decrease in Mental Support”). The current pandemic will incur adverse effects on all aspects of cancer care and negatively affect patients’ overall outcome. Collaboration among participating institutions, starting with dedicated work on cancer registries (an activity that has been negatively impacted by the pandemic at several major cancer centres, and specifically highlighted by Myanmar and Pakistan) would be beneficial to best address such major issues and limit the explosion of late-stage cancers and death following the pandemic. 

*Operations and Finance*

Apart from challenges in cancer care delivery, the COVID-19 pandemic has affected the operations and finances of many cancer centres. Workload was affected and reduced partly because of intentional rescheduling of patients but also partly due to fear of patients in seeking treatment for fear of being infected, especially since patients with cancer could be at an elevated risk of COVID-19 severe infection and mortality (Shankar et al., 2020). On the other hand, several institutions highlighted the additional need to allocate staff in COVID-19 specific wards, which disturbed the human resource management of the institutions.

Nine (53%) institutions (Bangladesh, India, Indonesia, Iran, Malaysia, Myanmar, Nepal, Pakistan and Singapore) reported shortages of medical staff, attributed mainly by diversion of staffs to COVID-19 care and COVID-19 infection in staffs. Shortage of supplies of drugs, gloves, masks, PPE were reported by 8 institutions (47%) (Bangladesh, Bhutan, India, Indonesia, Iran, Japan, Malaysia, Myanmar and Singapore. The impact on fundraising and income during the pandemic was also highlighted by 30% of participating institutions (India, Indonesia, Pakistan, Singapore and Vietnam) (Table 1).

Eight of the 17 participating institutions (47%) emphasized the impact of lockdowns on cancer care, including the prevention of infection transmission which made the movement of cancer patients difficult in view of travel bans (Hwang et al., 2021). Physical distancing norms were followed by most of the participating countries. In the early phase of the pandemic at time of paucity of data, the publications of various viewpoints, case reports, case series and opinion pieces helped most experts and institutions to optimize cancer care amidst COVID-19 (Kanesvaran et al., 2020; Kwek et al., 2021) through continued learning and evolution of our clinical practice. On top of these restrictions, international collaboration was heavily impacted as well, as pointed out by the national cancer centres of India, Iran, Japan, Korea, Malaysia, Mongolia, Pakistan, Singapore, and Vietnam (Table 1). 

Public communication on COVID-19 and Cancer is required to minimize fear and anxiety, so awareness and health promotion activities were required to reach the masses. Communication campaigns should be conducted to adequately inform citizens of the critical need to immediately visit their healthcare professional in case of suspected cancer symptoms and to maintain diagnostic and treatment schedules. Fifteen institutions (88%), mostly ANCCA members played the crucial role of disseminating reliable information to other centres nationwide. They highlighted how they managed to advertise and disseminate implications of COVID-19 and cancer care to the public In addition, 14 institutions (82%) managed to put in place or update guidelines or special protocols for cancer care in the pandemic (Table 1). In Myanmar, a national guideline was created on treatment prioritization as well as general and specific measures during COVID pandemic. Public health education on the importance of hand hygiene and wearing of face masks was also carried out (Hwang et al., 2021).

Cancer research and clinical trials were affected in most participating countries due to reduction in patient recruitment, funding restrictions, deployment of staff elsewhere and movement restrictions during lockdown (Singh and Prasad, 2020). A dramatic drop in new cancer drug trials amidst COVID-19 was reported in many countries (Dewi et al., 2020; Hwang et al., 2021; Wilkinson, 2021). 

In most participating major cancer centres, administrative teams were mobilized to help in providing operational support, which included priority access to COVID-19 testing for all cancer patients, installation of scanners at entry points, symptom screening of staff, patients and caregivers, restricted entry to visitors, as well as procurement of cancer medicines affected by shortages (Hwang et al., 2021). Protecting the safety and health of all healthcare providers is paramount and the procurement masks, PPE, and sanitizers was an absolute requirement for the delivery of quality cancer care. Operational teams in hospitals also helped to enforce protocols while creating additional special units and isolation facilities for COVID-19 positive patients (Hwang et al., 2021). 

All institutions were financially affected by the pandemic, for various reasons, including decrease of patient visits, treatments, change of treatment protocol, re-allocation of staff (a wide range of issues, ranging from extensive need such as in COVID-19 ward as well as a dramatic decrease of staff, such as screening activities). The sharing of the painful experience and countermeasures among participating cancer centres and countries is likely to create a virtuous cycle based on lessons learned to solve this second big question. 

*Will telemedicine and technological innovations revolutionize cancer care?*

A positive aspect that has emerged during the pandemic is the deployment of telemedicine to support cancer care across Asia (Yadav et al., 2020; Hwang et al., 2021) (participating institutions emphasized the use of video, telephone, and other electronic communication, including software for virtual tumour boards). Telemedicine and tele-health-based interventions have emerged as reasonably practical solutions to these impediments in the delivery of care to cancer patients (Grewal et al., 2021). Since most of the regular face-to-face visits for cancer patients have either been postponed or cancelled, tele-health-based interventions allow oncologists to take care of their patients remotely and monitor their progress. 

Many medical societies and physicians are adopting this option as the safest, both for the patient and the medical staff (Elkaddoum et al., 2020). With disruption in the cancer care delivery, teleconsultation helped oncologists and cancer patients to minimize the fear and anxiety with continuous communication (Hwang et al., 2021). All participating institutions (17/17, 100%) adopted tele-oncology practice to bridge the gap among specialists and, when feasible, between patients and clinicians in this challenging time (Table 2), when active treatment requiring in-person visits for a comprehensive clinical evaluation (in-person interview, physical examination, chemotherapy radiological and laboratory investigations) could be replaced by virtual consultations as a countermeasure to ensure safety of patients and medical staffs wherever possible as a response to the concrete challenges experienced by the majority of participating Asian cancer centres such as the reduction of cancer patient visits (due to reasons including patients under quarantine, travel restrictions, reluctancy of patients to travel) and the difficulties in procuring the cancer treatment (Table 1). 

Although the implementation of telemedicine may be difficult in resource limited settings, it can also allow technologically advanced cancer centres to ensure access to laboratory and pathology services to cancer patients even in remote areas during current times through establishment of satellite virtual care centres (Sirintrapun and Lopez, 2018). The recommended new prescriptions can be sent and filled locally, and participating cancer centres confirmed that permission was granted to use remote treatment and drug postal services to maintain the continuum of treatment whenever possible. The tangible application of telemedicine to manage difficult cases remotely may also include chemotherapy administered under guidance and monitored by remote supervision of cancer treatment as a new future norm. As part of it, several participating institutions raised the importance of an implementation or expansion of multidisciplinary team virtual meetings to ensure no delays in cancer diagnosis or treatment even for cases difficult to treat if not discussed among specialists (topic emphasized by Brunei and Myanmar, which started online meetings and case discussions with overseas counterparts). Not to be ignored is the fact that, as not all poor patients had smart phones or could schedule telemedicine appointments, this digital divide can be perceived as a barrier to access (Bakhtiar et al., 2020).

In order to provide care and support to cancer patients while avoiding unnecessary risks of infection, all health systems should set up strategies for the appropriate and proportional use of telemedicine in cancer care both during and after the pandemic period (Tuech et al., 2020). This should incorporate appropriate training opportunities for relevant healthcare professionals and expertly formed guidance on the best use of telemedicine in the cancer setting. Relevant regulations in the field of telemedicine should also be urgently defined to avoid unnecessary litigations. 

One major initiative, which is likely to expand and last is the focus on national referral networks, to improve cancer care through collaboration of specialists in institutions from urban and remote areas, already started by 7 institutions (41%) (Bangladesh, Brunei, India, Indonesia, Japan, Myanmar, and Singapore) (Table 2).

*Will Virtual conferences continue, at least in part, after the pandemic?*

With in-person cancer conferences curtailed during the Covid-19 pandemic, oncologists have struggled to continue the development and training they have historically relied on these meetings for. Face-to-face meetings have previously provided opportunities to interact with colleagues and experts, leading to many research and academic collaborations.

The worldwide impact of the pandemic has promoted the introduction and enhancement of virtual conferences, providing ample learning opportunities through participation to symposia and major conferences, while also allowing opportunities to collaborate through various smaller web-based meetings. Some unexpected benefits have included significant increase in conference attendance, with more participants from Asia who, previously, might have had difficulty attending in-person meetings due to geographic, or financial constraints. As clinicians and healthcare professionals from developing countries were the most impacted target of these essential learning opportunities to improve cancer care in these underserved regions, these virtual meetings have already started to positively impact the region (highlighted by participating institutions who were grateful for the new learning opportunity offered by the ANCCA-APOCP symposium on cancer care amidst COVID-19). Further, the virtual platform maximized the participation, engagement, and concrete interaction of participants through use-friendly interactive chat, Q&A functions not available with in-person meetings. 

The content of virtual meetings also offers easy access via advanced search mechanisms, allowing oncologists to tailor their education to their specific topics of interest or other relevant learning opportunities, with minimal impact on their work schedule. 

These benefits are balanced by new challenges such as coordinating a live virtual meeting across many time zones, technical difficulties before and during the meeting, difficulty of sustaining the attendees’ undivided attention. Ultimately, if not properly addressed, the learning commitment in virtual formats may not be as strong as it is in face-to-face meetings. Measures such as requiring participants to turn on their cameras, offering two-way interactive sessions, and facilitating frequent participation through direct questions to participants can help to an extent but fall short with large groups. Another major advantage already proposed by major virtual conferences is the “on-demand” feature, giving participants the opportunity to attend specific lectures repeatedly at their preferred time.

With these benefits and limitations in mind, we anticipate the future will encompass hybrid meetings represented by in-person meetings mixed with an optimal amount of virtual educational content. 

*How do we prepare for the next pandemic or International emergency as an International Community?*

The COVID-19 pandemic has created many unprecedented and unexpected challenges while also revealing opportunities to improve patient access to high-quality cancer care and research. COVID-19 vaccines have been discovered and are accessible to healthcare professionals as experienced by NCC Singapore and an aggressive vaccination program in the institution. There is a need for the participating countries in the Asian region to provide a roadmap of actions with the aim to help improve access to affordable and equitable care and clinical research. Streamlining excessive and unnecessary regulatory requirements in practice and research, as well as achieving improved outcomes for patients with cancer are also acute requirements that can be started to be addressed by participating institutions in the region (Pennell et al., 2021). 

Furthermore, there are still clear gaps in knowledge of Asian major cancer centres of COVID-19’s impact on cancer care that need to be addressed, in order to deliver successful cancer service recovery. These gaps include cancer treatment options, safety of cancer drugs, outcomes of COVID-19 treatments in patients receiving active treatment for cancer and precise impacts of the pandemic on cancer care (Di Lorenzo et al., 2020). 

To re-establish cancer care following the COVID19 pandemic, oncologists must remember the lessons learned from this pandemic and overcome the obstacles encountered in providing cancer care services. Clinicians, researchers, and academicians should be encouraged to share and publish their knowledge and findings on the impact of pandemic on cancer patients. It is important to understand the impact of modifications in cancer care delivery services and research during an emergency and adopt this knowledge as new standard practices to ensure safer, more effective, and higher quality care and research in the future and adapt to a new norm learned from this priceless Asian collaboration among key players in the field of cancer amidst COVID-19. ANCCA and APOCP have already started cooperation with City Cancer Challenge (C/Can), and the Union for International Cancer Control (UICC), aiming at increased access to quality cancer care (the “Quality Cancer Care” project) through multi-sector initiatives including virtual tumour board meetings with medical experts from C/Can, where an especially acute need has been observed in cities from LMICs which could have a greater burden of late stage presentation of cancer cases during and after the COVID-19 pandemic. 

Therefore, from clinical, moral, ethical and financial perspectives, healthcare providers including medical institutions, local working groups, payers, patients and decision-makers must collaborate and prioritize policy reforms to improve cancer care delivery service in the long term. This pandemic has increased our awareness about the shortcomings of the cancer care delivery system in Asia and taught us the importance of mitigating them by optimizing collaboration among cancer centres on establishing more cancer centres, especially in LMICs, to minimize disparities and inequalities in access to cancer care experienced by the general population.

**Table 1:** Challenges (As of 17 Mar 2021)

Country	Patient Care				Mental Impact		Oncology Management Impact		Administrative Impact					International Impact	Financial Impact
	Reduction of cancer patients’ visits	Difficulty or delay to procure the cancer treatment	Treating patients under quarantine	Increase of patients after the pandemics	Fear and anxiety of patients or mental support decrease	Fear and anxiety of staff	Delay or suspension of cancer prevention, detection or screening	Limited awareness and promotion activity	Medical HR shortage or infected staff	Shortage of supplies	Impact on regular work style	Movement restriction	Physical distancing	International collaboration	Fundraising & income
Bangladesh	✓	✓		✓	✓	✓		✓	✓	✓	✓	✓	✓		
Bhutan	✓	✓			✓	✓		✓					✓		
Brunei Dar.		✓	✓	✓			✓	✓							
India	✓	✓	✓	✓	✓	✓	✓		✓	✓	✓	✓	✓	✓	✓
Indonesia	✓	✓	✓	✓	✓	✓	✓	✓	✓	✓	✓	✓	✓		✓
Iran		✓	✓	✓	✓	✓	✓		✓	✓				✓	
Japan	✓	✓	✓				✓	✓		✓				✓	
Korea	✓				✓		✓						✓	✓	
Malaysia	✓		✓	✓		✓			✓	✓	✓	✓	✓	✓	
Mongolia	✓	✓				✓	✓	✓					✓	✓	
Myanmar	✓	✓	✓	✓	✓	✓		✓	✓	✓			✓		
Nepal	✓	✓	✓		✓				✓			✓			
Pakistan	✓	✓	✓	✓	✓	✓	✓		✓		✓	✓	✓	✓	✓
Singapore	✓	✓	✓	✓	✓	✓	✓		✓	✓	✓			✓	✓
Thailand	✓	✓		✓											
Taiwan			✓				✓								
Vietnam	✓	✓	✓	✓	✓		✓	✓			✓			✓	✓

**Table 2:** Countermeasures (As of 17 Mar 2021)

Country	Patient Care		Mental aspect	Oncology Management Countermeasures						Administrative Countermeasure			Finances and Research
	Virtual consultation / Telemedicine	Personnel and visitor management or restrictions	Psychological support / stress check	Guidelines / special protocols	Special unit	Isolation facility / quarantine separation	Advertisement / Dissemination	Triage / Priority change	Improving / implementing referral network	Introduction of scanner / detection	Procurement of sanitizing products and sanitation care	Remote work / work from home	Research institute
Bangladesh	✓	✓	✓	✓	✓		✓		✓	✓	✓		✓
Bhutan	✓	✓	✓	✓	✓		✓	✓		✓	✓		
Brunei Dar.	✓	✓	✓	✓	✓	✓		✓	✓	✓	✓		✓
India	✓	✓	✓	✓	✓	✓	✓	✓	✓	✓	✓	✓	
Indonesia	✓	✓	✓	✓	✓	✓	✓		✓	✓	✓	✓	✓
Iran	✓	✓		✓	✓	✓	✓	✓		✓	✓	✓	✓
Japan	✓	✓	✓	✓	✓	✓	✓	✓	✓	✓		✓	
Korea	✓	✓	✓	✓	✓	✓	✓	✓		✓	✓	✓	
Malaysia	✓	✓	✓	✓	✓	✓	✓	✓		✓	✓	✓	
Mongolia	✓	✓	✓	✓	✓	✓	✓	✓		✓			
Myanmar	✓	✓	✓	✓			✓		✓		✓	✓	✓
Nepal	✓	✓		✓		✓				✓			
Pakistan	✓	✓	✓	✓	✓	✓	✓	✓		✓	✓	✓	
Singapore	✓	✓	✓	✓	✓	✓	✓	✓	✓		✓	✓	✓
Thailand	✓	✓	✓			✓	✓			✓	✓		
Taiwan	✓	✓	✓		✓		✓						
Vietnam	✓	✓	✓		✓	✓	✓	✓		✓	✓	✓	✓

**Author Contribution Statement**

Laureline Gatellier (LG), Abhishek Shankar (AS) , LK Mela Dewi (MD), Quazi Mushtaq Hussain (MH), Tashi Dendup Wangdi (TD), Dato Babu Sukumaran (DB), Manju Sengar (MS), C S Pramesh (CSP), Nina Kemala Sari (NK), Sahar Tavakkoli Shiraji (ST), Mohammad Biglari (MB), Mamak Tahmasebi (MT), Satoshi Iwata (SI), Tatsuya Suzuki (TS), Seung-Kwon Myung (SM), June Young Chun (JYC), Jong Soo Han (JSH), Fen Nee Lau (FL), Suhana Yusak (SY), Luvsandorj Bayarsaikhan (LB), Khin Thin Mu (KTM), Kishore K. Pradhananga (KP), Aasim Yusuf (AY), Ching-Hung Lin (CL), RuRu Chun-Ju Chiang (RC), Suleeporn Sangrajran (SS), Quang Tien Nguyen (QN), Giang Huong Nguyen (GN), Aung Naing Soe (ANS), Tomohiro Matsuda (TM), Alireza Mosavi (AM), William Hwang (WH) LG, AS, MD, NK, TM, AM, WH conceived of the original project and presented idea. AS, AM developed the platform for meeting (symposium preparation and organization). LG, AS, MD, NK, TM, AM, WH contributed to gathering relevant stakeholders in the region. LG, AS, MD, MH, TD, DB, MS, CSP, NK, ST, MB, MT, SI, TS, SM, JYC, JSH, FL, SY, LB, KTM, KP, AY, CL, RC, SS, QN, GN, ANS, TM, AM, WH prepared country and institution challenges and countermeasures. SI, TS, TM, WH provided additional scientific review and evaluation of the symposium preparation and follow up discussions. theory and performed the computations. C.D. and D.E. verified the analytical methods. B.C. encouraged A.B. to investigate [a specific aspect] and supervised the findings of this work. All authors discussed the results and contributed to the final manuscript. LG, AS, MD wrote the manuscript with support from WH and AM, and input from all authors. WH and AM supervised the project. TM and TS helped supervise the project. LG, AS, MD, AM and WH contributed to the final version of the manuscript. All authors provided critical feedback and helped shape the research, analysis, and manuscript. 
